# Optimizing Feasibility and Acceptability of an Online Expressive Writing Intervention for Survivors of Adolescent and Young Adult Cancer: A Pilot Randomized Trial of Iterative Modifications and Outcomes

**DOI:** 10.1002/pon.70419

**Published:** 2026-03-03

**Authors:** Eunju Choi, Yusi (Aveva) Xu, Celia CY Wong‐Meli, Michael E. Roth, Yisheng Li, Qian Lu

**Affiliations:** ^1^ Department of Health Disparities Research The University of Texas MD Anderson Cancer Center Houston Texas USA; ^2^ Department of Nursing The University of Texas MD Anderson Cancer Center Houston Texas USA; ^3^ Division of Pediatrics The University of Texas MD Anderson Cancer Center Houston Texas USA; ^4^ Department of Biostatistics University of Texas MD Anderson Cancer Center Houston Texas USA

**Keywords:** adolescent and young adult cancer survivor, expressive writing, feasibility studies, FRAME‐IS, internet‐based intervention, intervention modifications, pilot randomized trial

## Abstract

**Objective:**

To evaluate the feasibility of an online expressive writing (EW) intervention for survivors of adolescent and young adult (AYA) cancer and determine whether iterative, theory‐driven modifications can enhance response, adherence, and completion rates.

**Methods:**

In this randomized pilot trial, survivors of AYA cancer were recruited through a hospital‐based AYA oncology clinic and an online community. Forty participants were randomly assigned to the EW intervention or control group. To improve on lower‐than‐expected adherence and completion rates in a previous cohort, the protocol was iteratively revised using the Framework for Reporting Adaptations and Modifications to Evidence‐based Implementation Strategies (FRAME‐IS). Modifications included extending the intervention from 3 to approximately 6 weeks, allowing more flexibility; two prompt choices per session; and proactive, personalized reminders. Response, adherence, and follow‐up survey completion rates at 1, 3, and 6 months were collected via REDCap surveys, and participant feedback was obtained through post‐study interviews.

**Results:**

We achieved a 46% response rate, compared to 40% in the previous cohort. Adherence improved significantly, with 83.3% of participants completing all 3 writing tasks versus 25.0% in the previous cohort (*p* < 0.001). The 6‐month follow‐up completion rate also increased to 72% from 50% in the previous cohort. Qualitative feedback indicated that the flexible timeline, tailored prompts, and personalized reminders were well received.

**Conclusions:**

Iterative, FRAME‐IS–guided modifications markedly improved the feasibility of the online EW intervention for AYA cancer survivors. These findings support further research to assess the clinical efficacy of EW in enhancing health outcomes and quality of life.

## 1 Background

Survivors of adolescent and young adult (AYA) cancer, defined as those diagnosed between ages 15 and 39, face profound psychosocial challenges shaped by their unique developmental stage [1]. AYAs navigate critical milestones such as identity formation, educational and career pursuits, and social relationships—tasks often disrupted by cancer [2, 3]. This population reports elevated rates of psychological distress, including anxiety and depression, compared to their healthy peers, alongside enduring feelings of social isolation and existential uncertainty about their futures [1, 4, 5]. Despite these vulnerabilities, scalable, developmentally tailored psychosocial interventions remain scarce. Many existing programs are resource intensive (e.g., in‐person therapy) or fail to align with AYAs' developmental priorities, digital preferences, and busy lifestyles, leaving a critical gap in accessible, tailored support. Prior research of such programs has reported low compliance and retention rates among AYAs, largely due to their hectic schedules and competing responsibilities [6, 7].

Expressive writing (EW)—a structured intervention encouraging emotional disclosure through written narratives—has demonstrated promise in mitigating distress among cancer survivors. Grounded in Pennebaker's paradigm [8], EW fosters cognitive and emotional processing through cognitive reappraisal and emotional regulation, enabling individuals to construct coherent narratives, reduce emotional inhibition, and derive adaptive meaning from cancer‐related challenges. Meta‐analyses link EW to reduced anxiety, improved quality of life, and lower healthcare utilization in oncology populations [9]. However, most EW studies have focused on older cancer survivors, neglecting AYAs' distinct needs. Few interventions are delivered online, and fewer incorporate developmentally appropriate content—an essential aspect of AYA survivorship [10]. Moreover, rigid EW protocols (e.g., fixed timelines, standardized prompts) may limit engagement among AYAs balancing treatment, work, education, and parenting.

To optimize EW interventions for AYA cancer survivors, iterative feasibility testing is essential. Adaptive designs allow researchers to refine protocols in response to participant feedback and logistical barriers, yet modifications are often poorly documented, hindering transparency and replication. We applied the Framework for Reporting Adaptations and Modifications to Evidence‐based Implementation Strategies (FRAME‐IS) to systematically report intervention changes [11] to enhance the feasibility of an online EW intervention for AYAs. Following lower‐than‐expected adherence and completion rates in a previous cohort [12], we iteratively revised the protocol by extending timelines, tailoring prompts, and adding proactive support to better align with AYA survivors' preferences and lifestyles. This study details how structured, theory‐driven modifications improved response, adherence, and completion rates, offering a roadmap for scalable, developmentally informed psychosocial interventions in oncology.

## 2 Methods

### 2.1 Study Design

This randomized pilot trial evaluated the feasibility of an online EW intervention for AYA cancer survivors that had been iteratively modified from the original protocol [12]. The study followed the CONSORT 2010 extension for pilot/feasibility trials [13], received ethics approval (Institutional Review Board Protocol #2020‐0538), and was registered at ClinicalTrials.gov (NCT04776941).

### 2.2 Participants

Eligible participants were 18 years or older at enrollment, had initially been diagnosed with cancer (any type and stage) between the ages of 15 and 39 within the past 3 years, had access to a digital device and internet connection, and were proficient in English. Recruitment strategies differed between cohorts. Between January and May 2023, participants were recruited by two main methods. First, research data coordinators identified eligible patients from a hospital‐based AYA oncology clinic and contacted them via email, text, and phone. This initial contact included a web link to an online eligibility screening survey. Second, a study flyer was posted to an online AYA cancer community managed by the study hospital. Interested individuals contacted the study team through methods listed on the flyer, such as a QR code, web link, text message, or phone call. The individuals were then asked to complete self‐reported eligibility questions, which research data coordinators used to confirm individuals' eligibility. Eligible patients were contacted by phone and told the study's purpose, planned activities, schedule, and the research team's contact details. Informed consent was then obtained electronically via text message or email. Due to the feasibility nature of this study, recruitment outcomes were inherently uncertain, and the sample size was based on participant availability rather than on power calculations, as the anticipated number of participants was too small for formal hypothesis testing [14].

### 2.3 Randomization

Forty participants were randomly assigned to either the EW intervention or control group at a 2:1 ratio, using a minimization technique [15]. The assignment was stratified according to 4 variables that may contribute to differences in psychosocial outcomes [16, 17]: time since diagnosis (< 12 vs. 12 months to 3 years), cancer stage (0‐II, III, or IV), cancer type (breast cancer vs. no breast cancer), and age at cancer diagnosis (18–24 years vs. 25–39 years). All participants and research data coordinators were blinded to group assignments.

### 2.4 Intervention Modifications Using FRAME‐IS

After randomization, participants completed an electronic baseline questionnaire. Within 1 week of finishing this questionnaire, they were provided with a link to their initial writing assignment. The intervention lasted roughly 6 weeks, with three writing tasks scheduled at 16‐day intervals. For each session, participants first read a brief, positive message that introduced the topic, and then selected one of two available writing prompts (Supporting Information S1: Table 1). They were instructed to write continuously for at least 15 min, without concern for grammar, spelling, or sentence structure. Participants could enter their responses directly into REDCap or write on paper and upload a photograph of their writing.

The current protocol included several modifications based on findings from the previous cohort, systematically implemented using FRAME‐IS (Table 1). First, the recruitment method was changed to improve recruitment diversity by integrating the hospital‐based AYA oncology clinic with online community outreach. Second, the intervention timeline was extended from 3 weeks to slightly over 6 weeks, with sessions spaced at 16‐day intervals. This timing/sequencing adaptation was implemented in response to feedback from the previous cohort, who reported struggling with deadline stress. Third, whereas the previous cohort received a fixed set of five intervention prompts and four control prompts throughout the study, the current cohort were provided two writing prompt choices per session, with options varying weekly. This adaptation aimed to enhance engagement by reducing cognitive load and increasing personal relevance. Similarly, to lower participant burden, the minimum required writing duration was reduced to 15 min per session. Fourth, proactive personalized reminders were delivered via text message and/or email by research coordinators, an adaptation targeting forgetfulness, accommodating AYAs' busy lifestyles.

**TABLE 1:** Cohort comparison and FRAME‐IS modification summary.

Parameter	Previous cohort	Current cohort	Rationale for modification	FRAME‐IS category	Observed impact
Recruitment method	Breast cancer registry	AYA clinic + community flyers	Enhance diversity	Contextual adaptation	Improved recruitment diversity in ethnicity, marital status, education, income, age at diagnosis, cancer type, and time since diagnosis
Session interval	3 weeks (weekly sessions)	Approximately 6 weeks (16‐day intervals)	Reduce deadline stress	Timing/sequencing	Adherence ↑ (25% → 83%)
Session duration	≥ 30 min/session	≥ 15 min/session	Reduce burden	Content/format	Adherence ↑ (25% → 83%)
Writing prompts (intervention)	Same 5 writing topics every week	2 writing topics every week, these options varying by week	Reduce burden and enhance engagement	Content/format	Adherence ↑ (33% → 84%)
Writing prompts (control)	Same 4 writing topics every week	2 writing topics every week, these options varying by week	Match with intervention group	Content/format	Adherence ↑ (11% → 82%)
Participant contact	Standard reminders	Personalized SMS/email reminders	Mitigate forgetfulness	Delivery personnel	Retention ↑ (50% → 72% at 6 months)

*Note:* Observed impacts reflect the current cohort improvements over the previous cohort.

Abbreviations: AYA, adolescent and young adult; FRAME‐IS, framework for reporting adaptations and modifications to evidence‐based implementation strategies.

### 2.5 Feasibility Outcomes

The feasibility metrics were response rate, intervention adherence rates, and follow‐up survey completion rates at 1, 3, and 6 months (Table 2). Completion of writing tasks and questionnaires was tracked using REDCap electronic surveys.

**TABLE 2:** Feasibility outcomes comparisons between previous cohort and current cohort.

Metrics	Definition	Group	Previous cohort	Current cohort	*p*
Response rate	Participants who responded to our contact attempts/participants contacted	All	40.00%	45.78%	0.375
Adherence rate	Participants who completed at least one writing task/participants who completed baseline survey	All	87.50%	91.67%	0.675
		Intervention	86.67%	96.00%	0.545
		Control	88.89%	81.82%	0.999
	Participants who completed at least two writing tasks/participants who completed baseline questionnaire	All	75.00%	86.11%	0.321
		Intervention	73.33%	88.00%	0.392
		Control	77.78%	81.82%	0.999
	Participants who completed all three writing tasks/participants who completed baseline questionnaire	All	25.00%	83.33%	**< 0.001**
		Intervention	33.33%	84.00%	**0.004**
		Control	11.11%	81.82%	**0.006**
Completion rate	Participants who completed the 1‐month questionnaire/participants who completed baseline questionnaire	All	58.33%	77.78%	0.487
		Intervention	66.67%	80.00%	0.457
		Control	44.44%	72.73%	0.362
	Participants who completed the 3‐month questionnaire/participants who completed baseline questionnaire	All	66.67%	75.00%	0.682
		Intervention	66.67%	76.00%	0.716
		Control	66.67%	72.73%	0.999
	Participants who completed the 6‐month questionnaire/participants who completed baseline questionnaire	All	50.00%	72.22%	0.140
		Intervention	60.00%	72.00%	0.498
		Control	33.33%	72.73%	0.175

*Note:* The bold values indicate statistical significance at *p* < 0.05.

### 2.6 Statistical Analysis

Descriptive statistics, including means and percentages, summarized feasibility outcomes. Group and cohort comparisons were conducted using independent *t*‐tests for continuous variables and Chi‐square or Fisher's exact tests for categorical variables, as appropriate. When at least one expected cell count was less than 5, Fisher's exact test was used. A *p*‐value of < 0.05 was considered statistically significant. In addition, semi‐structured post‐study interviews were conducted with a subset of participants to explore their experiences with the intervention.

## 3 Results

### 3.1 Participant Recruitment and Characteristics

Enrollment and completion data are summarized in the CONSORT flow diagram (Figure S1). A total of 166 AYA cancer patients/survivors were screened. Forty participants were enrolled and randomly assigned to either the intervention group (*n* = 27) or the control group (*n* = 13). The response rate was 45.8% (76/166), exceeding the 40.0% (44/111) response rate observed in the previous cohort [12]. There were no statistically significant baseline differences between groups for any demographic or clinical characteristics (Supporting Information S1: Table 2).

### 3.2 Adherence and Completion

Adherence to the intervention improved significantly in the current cohort compared with the previous cohort. In the current cohort, 83.3% of participants (30/36) completed all 3 writing sessions, whereas only 25.0% (6/24) in the previous cohort achieved full adherence (*p* < 0.001) (Table 2). Subgroup analyses revealed significantly higher adherence in both the intervention (84.0%) and control (81.8%) groups compared to the previous cohort (*p* = 0.004 and 0.006, respectively).

Completion rates also improved. In the current cohort, 77.8% (28/36) of participants completed the 1‐month follow‐up, in contrast to 58.3% (14/24) in the previous cohort. At 6 months, 72.2% (26/36) remained in the study, an increase from 50.0% (12/24) in the previous cohort. However, the differences in completion rates between cohorts at any time point were not statistically significant. The most common reasons for attrition included loss of contact and time constraints.

### 3.3 Participant Feedback

Post‐study interviews with participants indicated that the intervention was beneficial in reducing stress. One participant stated, “[the intervention] helped with our stress in multiple ways, and in the moment it was very enriching.” The writing prompts and inspirational quotes were well received, with one participant commenting that “[some of the writing prompts and quotes] really spoke to me and I really enjoyed them.” Participants also appreciated the structured but flexible nature of the intervention, noting that the adapted content and format provided clear guidance while remaining manageable. Several participants reported that the intervention inspired them to continue writing independently beyond the study period, as expressed by one participant: “I could do this for a whole year, or you know, rest of my life.” Additionally, the proactive communication, particularly the personalized reminders from research data coordinators, was consistently praised, underscoring the value of ongoing support in enhancing the engagement through the 6‐month study period, contributing to the overall satisfaction with the intervention. In fact, participants considered the tailored SMS reminders to be “nice” and “never annoyed,” and appreciated the balance of deadline flexibility and accountability.

### 3.4 Intervention Modifications and Impact

Modifications guided by the FRAME‐IS framework were systematically evaluated (Table 1). Extending the intervention timeline from 3 to 6 weeks (timing/sequencing adaptation) and refining the writing prompts (content/format adaptation) resulted in improved adherence, with rates increasing from 25.0% in the previous cohort to 83.3% in the current cohort. Additionally, the introduction of proactive reminders contributed to an increase of 22% points in 6‐month retention.

## 4 Discussion

This study evaluated the feasibility of an iteratively modified online EW intervention for AYA cancer survivors, demonstrating improved adherence and response rates in the current study compared to the previous cohort. The 46% response rate and 84% adherence rate for completing all three writing tasks in the current study compare favorably with similar psychosocial interventions for AYAs [18, 19, 20].

The modifications implemented in the current cohort addressed key barriers identified in the previous cohort. Extending the intervention timeline allowed greater flexibility for participants balancing treatment, work, and school. Providing two writing topic choices per session increased engagement by improving the personal relevance of the tasks and the autonomy of the participants, while proactive reminders enhanced adherence. These results underscore the importance of adaptability in digital psychosocial interventions for AYAs, particularly given their unique developmental and lifestyle needs.

### 4.1 Implications

Findings from this study suggest that online EW is a feasible, scalable intervention for AYA cancer survivors, offering a low‐cost alternative to in‐person psychosocial support. The structured modifications, providing increased flexibility and tailored engagement strategies, could be applied to other digital interventions aimed at enhancing health outcomes in AYAs. The strong adherence rates in the current cohort emphasize the value of proactive communication, structured and relevant prompts, and a flexible timeline, suggesting that digital interventions should incorporate personalized reminders, tailor content, and consider population realities to sustain engagement.

This study also highlights the importance of systematically documenting intervention adaptations using the FRAME‐IS framework. This approach enhances transparency and reproducibility, addressing a key gap in feasibility research. A larger‐scale randomized controlled trial is warranted to assess the intervention's clinical efficacy, particularly in reducing physical and psychological distress. Future studies should also explore individual differences in engagement, identifying subgroups that may benefit most from EW.

### 4.2 Limitations

The study's generalizability is constrained by its small sample size. Selection bias may also affect its validity, as the sample may overrepresent highly motivated and help‐seeking individuals. In particular, participants recruited through the study flyer online may have had a preexisting preference for online interventions, potentially influencing engagement levels.

## 5 Conclusion

Iterative redesign using FRAME‐IS markedly improved the feasibility of an online EW intervention for AYA cancer survivors, with intervention adherence and follow‐up survey completion rates doubling between cohorts. These findings underscore the transformative potential of adaptive, developmentally informed interventions in psycho‐oncology. By centering AYAs' unique needs—flexible timelines, relatable content, and digital engagement—this study advances a scalable model for a population often marginalized in cancer care. Future research should build on these insights to bridge the gap between survivorship and thriving in AYA oncology.

## Author Contributions

**Eunju Choi:** conceptualization, data curation, formal analysis, investigation, methodology, project administration, visualization, writing – original draft, and writing – review and editing. **Yusi (Aveva) Xu:** writing – original draft and writing – review and editing. **Celia CY Wong‐Meli:** writing – review and editing. **Michael E. Roth:** resources and writing – review and editing. **Yisheng Li:** formal analysis, software, and writing – review and editing. **Qian Lu:** conceptualization, funding acquisition, investigation, methodology, project administration, resources, supervision, and writing – review and editing.

## Funding

Support for this work was derived (in part) from the annual distributions of the Permanent Health Fund endowment received by The University of Texas MD Anderson Cancer Center from the state legislature. Eunju Choi has received award number K99CA293336 from the National Cancer Institute/National Institutes of Health. However, this study was not directly funded by the grant.

## Conflicts of Interest

The authors declare no conflicts of interest.

## Supporting information


Supporting Information S1

**Figure S1:** CONSORT flow diagram.

## Data Availability

The data that support the findings of this study are available from the corresponding author upon reasonable request.
