# Supplementary material for: Optimizing Feasibility and Acceptability of an Online Expressive Writing Intervention for Survivors of Adolescent and Young Adult Cancer: A Pilot Randomized Trial of Iterative Modifications and Outcomes
Source: Psychooncology. 2026 Mar 3;35(3):e70419. doi: 10.1002/pon.70419 (PMC12954469; doi:10.1002/pon.70419)
Supplement: Supplementary file 1 — Supporting Information S1 [file PON-35-e70419-s002.docx]

| **Supplemental Table 1. Positive messages and writing topics** | | | | |
| --- | --- | --- | --- | --- |
|  | **Intervention group** | | **Control group** | |
|  | **Positive message** | **Writing topic** | **Neutral messages** | **Writing topics** |
| Week 1 | A compilation of three inspirational quotes about overcoming cancer | 1. Deepest thoughts and feelings about most stressful experiences 2. How you handled most stressful experiences and learned from them | Healthy eating | 1. Writing about your eating patterns 2. Your activities throughout the day |
| Week 2 | A short story about responding to adversity | 1. Appreciation for some aspects of life that you had not seen or expected 2. Any positive thoughts and feelings about your experiences and a re-evaluation of your life's meaning | Physical activity | 1. Describing your hobbies 2. Your activities throughout the day |
| Week 3 | A poem about “what cancer cannot do” | 1. Imagining an ideal future where all life dreams have been realized 2. Providing advice to someone undergoing similar struggles | Getting organized | 1. Describing your house 2. Your activities throughout the day |

| **Supplemental Table 2.** **Demographic and Clinical Characteristics of the Enrolled Sample at the Time of Consent (N = 36)** | | | | | | |
| --- | --- | --- | --- | --- | --- | --- |
|  |  | Group | | | |  |
|  |  | Intervention  (*n* = 25) | | Control  (*n* =11) | | *p* |
| Age, years, mean, standard deviation | | 32.00 | 6.16 | 30.73 | 5.83 | 0.566 |
| Sex at birth, *n*, % | |  |  |  |  |  |
|  | Male | 6 | 24.0 | 2 | 18.2 | 1.000 |
|  | Female | 19 | 76.0 | 9 | 81.8 |  |
| Marital status, *n*, % | |  |  |  |  |  |
|  | Never married | 16 | 64.0 | 5 | 45.5 | 0.465 |
|  | Married | 9 | 36.0 | 6 | 45.5 |  |
| Race, *n*, % |  |  |  |  |  |  |
|  | Black/African American | 1 | 4.0 | 0 | 0 | 0.516 |
|  | Asian | 4 | 16.0 | 2 | 18.2 |  |
|  | White | 19 | 76.0 | 7 | 63.6 |  |
|  | Native American | 0 | 0 | 1 | 9.1 |  |
|  | Other | 1 | 4.0 | 1 | 9.1 |  |
| Ethnicity, *n*, % | |  |  |  |  |  |
|  | Hispanic | 8 | 32.0 | 5 | 45.5 | 0.475 |
|  | Non-Hispanic | 17 | 68.0 | 6 | 54.5 |  |
| Education, *n*, % | |  |  |  |  |  |
|  | High school graduate | 2 | 8.0 | 0 | 0 | 0.287 |
|  | Some college or associate's | 2 | 8.0 | 1 | 9.1 |  |
|  | College graduate | 13 | 52.0 | 3 | 27.3 |  |
|  | Graduate or professional school | 8 | 32.0 | 7 | 63.6 |  |
| Annual household income, *n*, % | |  |  |  |  |  |
|  | Less than $15,000 | 7 | 28.0 | 2 | 18.2 | 0.757 |
|  | $15,000 to less than 45,000 | 5 | 20.0 | 2 | 18.2 |  |
|  | $45,000 to less than $75,000 | 5 | 20.0 | 4 | 36.4 |  |
|  | $75,000 or more | 8 | 32.0 | 3 | 27.3 |  |
| Age at diagnosis, *n*, % | |  |  |  |  |  |
|  | 18-24 years | 6 | 24.0 | 2 | 18.2 | 1.000 |
|  | 25-39 years | 19 | 76.0 | 9 | 81.8 |  |
| Time since diagnosis, *n*, % | |  |  |  |  |  |
|  | less than 12 months | 7 | 28.0 | 2 | 18.2 | 0.690 |
|  | 12 months to 3 years | 18 | 72.0 | 9 | 81.8 |  |
| Cancer type, *n*, % | |  |  |  |  |  |
|  | Solid | 20 | 80.0 | 10 | 90.9 | 0.643 |
|  | Hematologic | 5 | 20.0 | 1 | 9.1 |  |
|  | | | | | | |
