# Supplementary material for: Optimizing Feasibility and Acceptability of an Online Expressive Writing Intervention for Survivors of Adolescent and Young Adult Cancer: A Pilot Randomized Trial of Iterative Modifications and Outcomes
Source: Psychooncology. 2026 Mar 3;35(3):e70419. doi: 10.1002/pon.70419 (PMC12954469; doi:10.1002/pon.70419)
Supplement: Supplementary file 2 — Figure S1: CONSORT flow diagram. [file PON-35-e70419-s001.docx]

**Supplemental Figure 1. CONSORT Flow Diagram**

Assessed for eligibility (*n* = 166)

Excluded (*n* = 126) due to

♦  Not meeting inclusion criteria (*n* = 3)

♦  Declined to participate (*n* = 33)

♦  No response, could not be reached, or became unresponsive (*n* = 90)

Assessed at 3-month follow-up (*n* = 19)

Assessed at 1-month follow-up (*n* = 20)

Allocated to intervention (*n* = 27)

♦ Received allocated intervention (*n* = 25)

♦ Did not receive allocated intervention (did not complete baseline survey) (*n* = 2)

Assessed at 1-month follow-up (*n* = 8)

Allocated to control (*n* = 13)

♦ Received allocated intervention (*n* = 11)

♦ Did not receive allocated intervention (did not complete baseline survey) (*n* = 2)

Assessed at 3-month follow-up (*n* = 8)

## Allocation

## 1-Month Follow-Up

Randomized (*n* = 40)

## Enrollment

## 6-Month Follow-Up

Assessed at 6-month follow-up (*n* = 18)

Assessed at 6-month follow-up (*n* = 8)

## 3-Month Follow-Up
